# Deep Vein Thrombosis in Intravenous Drug Users: An Invisible Global Health Burden

**DOI:** 10.7759/cureus.18457

**Published:** 2021-10-03

**Authors:** Nidhi Jain, Chaithanya Avanthika, Abhishek Singh, Sharan Jhaveri, Ivonne De la Hoz, Gashaw Hassen, Genesis P Camacho L, Keila G Carrera

**Affiliations:** 1 Medicine and Surgery, Himalayan Institute of Medical Sciences, Dehradun, IND; 2 Internal Medicine, Sir Ganga Ram Hospital, Delhi, IND; 3 Hematology and Oncology, Brooklyn Cancer Care, Brooklyn, USA; 4 Paediatrics, Karnataka Institute of Medical Sciences, Hubli, IND; 5 Internal Medicine, Mount Sinai Morningside, New York, USA; 6 Internal Medicine, Smt. Nathiba Hargovandas Lakhmichand Municipal Medical College, Ahmedabad, IND; 7 Internal Medicine, Universidad del Zulia, Maracaibo, VEN; 8 Medicine and Surgery, University of Parma, Parma, ITA; 9 Medicine, Addis Ababa University, Addis Ababa, ETH; 10 Progressive Care Unit, Mercy Medical Center, Baltimore, USA; 11 Division de Estudios para Graduados, Facultad de Medicina, Universidad del Zulia, Maracaibo, VEN; 12 Gastroenterology, Universidad de Oriente (VEN), Maturin, VEN

**Keywords:** pwids (people who inject drugs), deep vein thrombosis management, dvt diagnosis, wells dvt score, venous thromboembolism (vte), injecting drug users (idu), intravenous drug use (ivdu), intravenous drug abusers (ivda), intravenous drug user, deep vein thrombosis (dvt)

## Abstract

The prevalence of intravenous drug use has increased in the past decade and it represents an important risk factor for deep vein thrombosis. Intravenous drug use is a global problem, with the main culprit being heroin. Peer pressure and poverty in high-risk groups such as sex workers, females, and young adults raise the risk of intravenous drug use, which expresses itself in the form of venous thromboembolism eventually. Deep vein thrombosis typically manifests itself eight years after the initial intravenous drug administration, rendering it a silent killer.

Aiming to review and summarize existing articles in this context, we performed an exhaustive literature search online on PubMed and Google Scholar indexes using the keywords “Deep Venous Thrombosis (DVT)” and “Intravenous Drug Users (IVDU).” English articles that addressed epidemiology, pathogenesis, clinical manifestations, diagnosis, differential diagnosis, management, and outcomes of DVT, including those in IVDU, were selected and analyzed.

The pathogenesis of DVT development in IVDU is mainly attributed to the interplay of trauma to the vessel by repeated injection and the injected drug itself. The right-sided femoral vein is the most common vein affected. Prevalent clinical presentations include local pain, swelling, and redness with typical systemic symptoms including fever, cough, dyspnea, and chest pain on top of addiction features. There appeared to be a delay in reporting symptoms, which was most likely due to the social stigma attached to IVDU.

There are over 50 conditions that present with swollen and painful limbs comparable to DVT in IVDU, making precise diagnosis critical for timely treatment. Venous ultrasound is the method of choice for diagnosing DVT. Extended anticoagulant therapy with low-molecular-weight heparin combined with warfarin is the recommended treatment. Intravenous drug abusers having DVT are affected by multiple complications and poorer outcomes such as slower recovery, recurrent venous thromboembolism (VTE), and a longer hospital stay, which put them at higher risk of morbidity, mortality, reduced productivity, and economic burden.

## Introduction and background

Intravenous drug use has become a major public health problem, the prevalence of which has increased significantly in the last decade. There are approximately 15.6 million people who inject drugs (PWID) worldwide and this may be an underestimate of the real problem as the use of injection drugs is an illegal and stigmatized practice, making data collection challenging [1,2]. Injection drug use and particularly sharing needle practices are known risk factors for skin and soft tissue infections, infective endocarditis (IE), and also blood-borne infections, which are a topic of concern having played an important role in the further spread of HIV and hepatitis B and C [2-5].

Since the 1990s, it has been demonstrated that vascular complications including deep vein thrombosis (DVT) have high morbidity among intravenous drug users (IVDU). Retrospective research conducted on 734 opioid users reported a rising risk of DVT and its complications among them, especially in women with increasing age, emphasizing the need for due vigilance on the part of primary care physicians and the development of effective DVT prevention and management strategies in IVDU [6]. A study conducted in Glasgow by McColl et al. showed that 52.8 % of women under 40 years of age with confirmed DVT were IVDU and similar results were obtained by Syed et al. who found this association in 48.8% of patients from a similar sample [7,8]. Although knowledge of the exact mechanism behind this association is still limited, repeated trauma to the vein may be partly responsible along with the intrinsic properties of the injected substances and the acidity of some preparations [6-10].

The clinical presentation of DVT in IVDU may differ from the presentation in the rest of the population. The incidence is higher in younger patients, and in terms of location, proximal vein involvement is more likely, with an even greater risk in those who inject drugs directly into the femoral vein, supporting venous injury as an important trigger for the development of DVT [6,9]. However, it is important to note that IVDU may have other risk factors that contribute to the development of DVT, including immobility, which may be an effect of the injected drugs, and also smoking, which is a common practice in this group [11]. There is controversy, however, as smoking has been reported as a risk factor for DVT in many studies [12], while in others, such a result is not stated [13].

In addition, IVDU with DVT are admitted more frequently and hospital stays tend to be longer, perhaps due to severe and proximal presentations, but also due to behavioral problems and lack of support system, which complicate diagnosis and management in this group and ultimately place a greater burden on healthcare costs [6,14-15]. Non-sterile needle use often leads to septic DVT, which exacerbates the problem and carries the risk of sepsis, IE, and septic pulmonary embolism, contributing to an overall poorer prognosis [15]. In addition, studies have shown that mortality for pulmonary embolism (PE) is even higher in IVDU who develop this complication than in non-IV drug users [7]. Recurrences within the first six months are more common in IVDU, with non-adherence to treatment playing an important role, and as most patients are lost to follow-up, information on long-term complications is lacking [15].

The increasing prevalence of injection drug use has contributed to a higher incidence of DVT in young patients and its association is undeniable [11]. However, this association may be overlooked by physicians, and intravenous drug use is not even included in the Wells criteria, the most commonly used tool to screen patients for DVT. Although research is challenging, more studies are needed to gain a better understanding of the problem. Broader knowledge will lead to the development of more appropriate preventive measures, diagnostic approaches, and treatment protocols [9,15].

## Review

Epidemiology

According to the United Nations (UN), approximately 269 million people used drugs worldwide in 2018 [16]. With a 20% increase in statistics since 2010, there has been a significant impact on the UN Sustainable Development Goals, which include within its objectives "to ensure healthy living and promote wellness for all at all ages" [17]. It is estimated that by 2030, global drug abuse will have increased by 11%, with Africa seeing an increase of up to 40%, reflecting at the same time a general increase among low- and middle-income countries and a decrease in developed countries. Much of this change is the result of demographic shifts in the population curve, changes in public policy, and legislative changes in many countries [18].

The most commonly used substances are alcohol, tobacco, and drugs such as cannabis; however, there are different patterns of abuse depending on the situation in each country. The real risks of drug abuse are ignored voluntarily or involuntarily by the general population, particularly by the younger generation, who are unaware of the effects of drug potency and the harm it can cause [19]. The use of drugs is frequently linked to the modern life, geographical location, and demographic characteristics of each population [20]. In addition to having higher morbidity, intravenous drug abusers have been proven to have a much higher death rate than the general population [21].

Many factors, such as poverty, curiosity, peer pressure, the existence of psychiatric pathology, and other psychosocial causes may lead people to use drugs, including intravenous drugs. Peer pressure and education level are the two social factors that highly influence drug use [6]. Although experimental use of illicit drugs like marijuana may begin between the ages of 12 and 13 in developed countries, in general, drug use worldwide is mainly distributed among people between the ages of 15 and 34, who, according to the UN, constitute the population most at risk for the decade [16,22].

Economic access is also a factor in intravenous drug abuse. Marijuana, inhalants, and cocaine are the most used drugs in developing countries such as Peru, and all drugs except marijuana can be administered intravenously [22]. Abusers often have a difficult time estimating how much of the substance they are injecting into their system because of the quick onset of the high and the intensity of the symptoms [23]. The search for a rapid increase in the concentration of drugs in the blood is the primary cause of intravenous drug abuse. Furthermore, many users crush tablets of one or more drugs for use as an injectable solution, and when doing so, they also inject the drug's adjuvants, which carry health risks independent of the ones from the active ingredient from which they seek pleasure [24]. Opioids are the most used intravenous drugs; in fact, heroin is the most prevalent drug among intravenous drug users [25].

Prescription pain relievers were misused by 9.7 million people in the United States by 2019. Despite a decrease from the peak observed in 2011, and from overall opioid use statistics of consumption that were particularly high from 2002 to 2018, these numbers remain high. In 2019, there was a decrease in opioid use across all age groups, particularly among adolescents [26].

Urbanism is another major risk factor for substance abuse. Although no studies have quantified the relationship between urbanization and drug use, it is a fact that urban conditions such as urban sprawl, low economic levels, overcrowding, unemployment, and crime rates have generated a positive correlation between urbanization and drug use. It is projected that the urban population will increase by approximately 23% during this decade, distorting the relationship between urbanization and drug use [27].

Another important factor complicating the achievement of this UN goal is an increase in the production of stimulants with different precursors than ephedrine and phenylephrine, with increased production being found in countries where other drugs were traditionally manufactured for trafficking, such as Mexico. Because of easier access, the redistribution of stimulant substances’ production increases the risk of their use by IVDU in middle and low-income countries [26,28].

People with socioeconomic disadvantages are at greater health risks when abusing substances, especially when using intravenous drugs, first because they are more likely to use drugs that are considered of lower quality or impure, and second, because a low income may lead to a higher risk of using drugs with syringes used by others or by themselves multiple times, increasing the radius of bacteria exposure, impurities in products, dangerous adjuvants in solid components of the drug, use of broken syringes, and parenterally transmitted diseases. These dangers are part of the economic constraints, low cultural level, or peer-pressure situation that can lead to intravenous drug use and cause a variety of pathologies, including DVT [29-31].

Controlled medical substances, which are part of the early drugs of abuse and are usually used for various medical problems such as pain and anxiety, are frequently found among the intravenous drugs that are intended to be diluted as an injectable solution [25]. In this regard, low-income countries face a shortage of opioids, whereas high-income countries have more than 90% of all opioids available to the world population, resulting in an increase in intravenous opioid use in these countries' substance-abusing populations [31].

Deep vein thrombosis is common among IVDU, with some studies estimating it to be as high as 13.9%. It is also reported that female IVDU are more likely to develop deep vein thrombosis, and as with any case of DVT, age is a major risk factor within this group, but there is a difference from non-IVDU because the mean age for developing DVT in the IVDU group is 30 years, which is a relatively young age in comparison with people from the same age and no known pathologies. Venous thromboembolism in IVDU occurs on average eight years after the intravenous drug is started, and heroin remains the main drug used [6,8].

Intravenous drug abuse can increase the risk of developing leg ulcers, secondary or after the clinical presentation of DVT by up to 15.7%, and it is thought to be in close relationship with a decrease in blood flow to the area that is injected [6,32]. In general lines, most authors correlate DVT in IVDU to the frequent trauma caused by injections, having as consequence constant damage to the endothelium and the release of tissue factor that sometimes is attenuated for the development of thrombophlebitis in the area [6].

Among the IVDU, sex workers have the highest risk for developing DVT, and this is related to the fact that they typically use high amounts of stimulants that usually have a very low pH and cause a stronger sympathetic reaction at the site of injection. Long-term problems are common in IVDU, and DVT may occur several times in every user, with an increased risk for septic DVT. Other common complications are post-thrombotic syndrome, which can occur in up to 50% of patients with DVT and chronic thromboembolic pulmonary hypertension that might be present in 3.3% of patients with other risk factors besides being an intravenous drug abuser and that usually presents after 10 years of having the first episode of DVT. These clinical manifestations affect the patients by lowering quality-of-life scores when compared to the general population and by increasing their rates of mortality [6,33-36].

Pathogenesis

DVT in IVDAs is a well-documented phenomenon [11,37]. The degree of vessel damage and venous insufficiency varies depending on a host of other risk factors. Vascular complications of IVDAs usually develop only after prolonged drug use. Due to superficial thrombophlebitis, long-term users inject the drug directly into deep veins [38]. Some candidates include the groin, deep femoral vein, and even the superficial orbital vein [39-40].

The mechanism of DVT in the general population is a matter of debate. Authors have proposed different mechanisms such as the increased propensity of coagulation, valvular damage due to anoxia, and stasis [7]. The association of DVT development in IV drug abusers is mainly because of the interplay between two independent factors working together.

Injected Drug

Cornford et al., in a seminal case-control study conducted in the UK, established that crack and cocaine were significantly associated with DVT, whereas heroin and amphetamines were negatively and independently associated with DVT. Moreover, no statistically significant difference was found between the injection frequency of the drug and the development of DVT [6]. This was in line with existing literature that described "Virchow's triad" and increased platelet aggregation as being one of the proposed mechanisms underlying cocaine-associated DVT [41].

Another theory explains this association of IV drug use and DVT to be due to the citric acid used to dissolve these drugs. Most heroin available reaches a pH of nearly 4, which is quite low as compared to the normal pH within blood vessels [6,33-34,42].

Injection of the Drug

Most authors suggest that it is the repeated puncture of the vein, consequently leading to endothelial damage and release of tissue factors that is responsible for DVT. Moreover, the coincidental infection and superficial thrombophlebitis further attenuate this risk [8,43].

In a case-control study conducted in Iran, Masoomi et al. found significant differences in opioid users depending on the method of drug usage (oral, inhalation, injection, etc.) [44]. Compared to other methods, the injection method increased the risk of subsequent DVT sixfold. Moreover, they found that opioid addiction per se was not a significant risk factor for DVT, but have attributed this to a limitation in the size of the study [44].

Other supplementary mechanisms have also been proposed. For instance, drug addiction is an important risk factor for a patient’s immobility. Intravenous drug abusers are known to be in prolonged states of inactivity during and after use thus further propagating the risk of DVT [43]. Moreover, the reduced blood flow resulting from inactive muscle pumps causes anoxic damage and further increases the DVT risk [8,43]. Increased levels of coagulation factors due to infections introduced from injections may also contribute to DVT [8]. Because of venous deterioration, diagnostic procedures are further complicated [7].

Clinical manifestations

In the subpopulation of IVDU, the most common vein to be affected was found to be the femoral vein, followed by the iliac vein. Most often, it is presented either right-sided or bilateral [15]. Bilateral thrombosis is estimated to occur 6.8% to 37% of the time; however, thrombosis in the opposite leg might be asymptomatic. The most commonly occurring local symptoms were groin pain, swelling, erythema, elevated local temperature, and/or cyanosis or skin necrosis of the affected limb [15].

Systemically, a typical IVDU with DVT presents with fever, cough, dyspnea, and chest pain associated with other symptoms of addiction [15]. It is reasonable to assume that the medical staff caring for patients with a history of intravenous drug use will encounter, in addition to the clinical presentation of the main disease, addiction symptoms, side effects of the drugs' toxic effects, psychiatric symptoms, and sociological issues such as poor hygiene, a negative attitude toward health care and/or therapy, homelessness, prostitution, lack of insurance, and so on [45].

The typical age of a first (or only) DVT for an intravenous drug abuser was 30 years, and it happened on an average of eight years after the first injection drug was taken. Although the risk of getting a pulmonary embolism (PE) looked to be 2-6%, 15.7% of those who had previously experienced DVT developed leg ulcers, and some experienced additional problems [6]. In a study conducted by Thokchom et al., one-third of the 109 individuals who presented to an emergency room with a suspected DVT were IVDU [46].

Patients seeking treatment for drug addiction who had previously had a DVT reported a lower subjective physical and mental health status than those who had not previously experienced a DVT [6]. The significant frequency of long-term limb problems and patients with previous DVT having worse health and well-being scores is a cause for grave concern. Leg ulcers are more likely to be correctly reported since they require nursing treatment, while other problems and post-thrombotic complications are more likely to be inaccurate and under-reported [6].

When it came to responding to symptoms, there was usually a lag. One reason was the stigma, which will be discussed in further detail later, but several patients hesitated because they were afraid of amputation or death. Several patients stated they were unaware it was a DVT, and a couple blamed it on a "missing" injection at first [47]. DVT has a wide range of long-term symptoms and physical consequences. Pain, as well as swelling, were commonly mentioned discomfort in the leg, and for a couple, pain all over the body, was another reason for IVDU to revert to opioids, to manage the pain [47].

A study by Girard et al. found more than half of patients affected by DVT of proximal veins (such as iliac, femoral, and popliteal) have a concurrent presentation with PE, which is a form of venous thromboembolism (VTE) with a variable presentation that needs urgent management (both diagnosis and treatment) to reduce fatality [48].

Diagnosis

Not only is it crucial to diagnose deep vein thrombosis as soon as possible to avoid fatal consequences such as PE, but it is also important to avoid needless anticoagulation in the absence of a thrombus. Any clinical suspicion of phlebothrombosis should be confirmed by objective tests, as even minor symptoms might mask more serious disease, whereas the characteristic signs of leg swelling and discomfort can also be associated with non-thrombotic conditions [49-54].

Although generic, the history and physical exam are important parts of the diagnostic process because they help rule out the possibilities and identify patients as having a low, intermediate, or high risk of venous thrombosis. Wells et al. developed a simple clinical scoring method based on three factors: signs and symptoms at presentation, the presence or absence of risk factors, and the possibility of an alternative diagnosis, demonstrating that approximately 80% of people with a high probability clinical score have venous thrombosis, while only 5% of people with a low probability clinical score have venous thrombosis. Pretest probability scores can be used in conjunction with noninvasive testing to save costs and simplify the diagnostic process [55-56].

Testing modalities

Three reliable tests for diagnosing DVT in patients with symptoms are impedance plethysmography, venous ultrasonography, and venography, with venous ultrasonography (also known as B-mode imaging) being the most accurate [57-83]. A D-dimer test (SimpliRED) performed on a patient's finger-prick blood sample is also adequate to rule out DVT. However, the D-dimer test produces false-positive results after trauma or surgery, limiting its application in these conditions [84]. Due to the unreliability of clinical diagnosis of DVT, Tovey et al. advocated plethysmographic techniques and D-dimer tests as screening investigations and venography or ultrasonography as confirmatory tests for diagnosing DVT [85]. Arumilli et al. also highlighted that depending simply on D-dimer levels for diagnosis could be very misleading because these levels can be moderately raised in other disorders as well. Moreover, any delays in diagnosis may adversely influence the prognosis, thus rapid imaging or investigations should be prioritized [86]. Based on a meta-analysis of studies performed between 1970 and 2009, Johnson et al. concluded that a single negative whole-leg compression ultrasonography (CUS) excludes both proximal and distal DVT, and that repeat CUS is not required to rule out DVT [87]. Per a study done by Geersing et al., a low Wells score paired with a negative D-dimer test can rule out DVT. However, this does not hold for cancer patients. Whereas for those with suspected recurrent DVTs, adding one point to the rule allows for a safe DVT exclusion [88]. Lately, two-point and three-point point-of-care ultrasound (POCUS) procedures have come up as excellent tools for diagnosing DVT in the ED, displaying high specificity and sensitivity, especially when administered by a POCUS-trained emergency physician [89]. However, a 10-minute imaging procedure called magnetic resonance direct thrombus imaging (MRDTI) has been found to be superior to compression ultrasonography and capable of discriminating between acute and chronic thrombosis remnants. Withholding anticoagulation after a negative MRDTI scan resulted in a failure rate of 1.7% (95% CI), which was lower than the 6.5% predefined safety threshold value, proving that MRDTI was a simple, reproducible, and feasible test for diagnosis of recurrent venous thromboembolism [90]. For ruling out suspected DVT in pregnancy, ultrasound proves to be an effective diagnostic test, based on a study revealing that a single negative ultrasound scan had a low false-negative rate [91]. In 2020, Kraaijpoel and his team demonstrated that all three imaging techniques, single limited vs. serial limited vs. whole-leg CUS, have equal failure rates. However, the relative failure rate of a single restricted CUS was unclear, making it only suitable for low-risk applications [92]. CUS was also recommended as the preferred diagnostic method in cancer patients by Takada et al. [93]. Conversely, Palanisamy et al. supported the effectiveness and convenience of Wells predictive score for identifying DVT in the general population well in time to commence anticoagulation without delay [94]. Latest studies have shown that the new imaging technique, Delay Alternating with Nutation for Tailored Excitation black-blood preparation combined with a Fast Low-Angle Shot (DANTE-FLASH) produced superior picture quality and detected thrombi far more accurately than MRDTI and ultrasound, recommending it as a practical, time-efficient, and safe option to diagnose DVT [95].

The updated European Society of Cardiology 2021 guidelines on acute DVT diagnosis and management recommend using a clinical prediction rule, such as the modified two-level Wells score, for stratification of patients with DVT suspicion, employing immunoturbidimetric tests (high sensitivity) or enzyme-linked immunosorbent assay (ELISA) D-dimer assays to rule out DVT in patients with an "unlikely" clinical probability of having DVT, venous ultrasonography (US) as the method of choice for diagnosing DVT, CT venography to be reserved for those with suspected upper extremity DVT having a negative venous ultrasound, and considering a venous ultrasound for further severity stratification in patients suspected of having concurrent PE [96]. Recently, Di Minno et al. discovered a significant association between a positive D-dimer test after stopping oral anticoagulation and recurrent VTE, while restarting oral anticoagulation after a positive D-dimer test was found to be inversely linked with VTE recurrence [97].

Differential diagnosis

Table 1 summarizes all the possible differentials of deep vein thrombosis.

**Table 1:** Differential diagnosis of a painful, swollen limb. References [90,91,98-102].

System	Differential diagnosis
Musculoskeletal	Muscle strain or tear, arthritis, Achilles tendonitis, ruptured muscle or tendon, trauma or twisting injury to the leg, calf hematoma, ruptured Baker’s cyst, psoas or gluteal abscess, heterotrophic ossification, cellulitis, internal disruption of the knee, fracture
Gastrointestinal and hepatobiliary	Femoral hernia, liver cirrhosis, Budd-Chiari syndrome, retroperitoneal fibrosis
Cardiovascular	Deep vein thrombosis, post-thrombotic syndrome (especially lipodermatosclerosis and venous eczema), arterial aneurysms, cutaneous vasculitis, arteriovenous fistula, changes in wall lumen (aplasia, intraluminal spurs), acute arterial ischemia, peripheral arterial disease, chronic venous insufficiency, varicose veins, venous obstruction, superficial or septic thrombophlebitis, congenital vessel abnormalities
Lymphatic	Lymphedema, lymphadenopathy, lymphangitis, lymphatic obstruction
Renal	Nephrotic syndrome, nephritic syndrome, renal failure
Nervous	Swelling in the paralyzed leg (stroke)
Tumors	Lipoma, intraluminal or vascular wall tumor, malignancy – sarcoma, liposarcoma, carcinoma, lymphoma

Management

Screening

Injection drug users are at a greater risk of DVT, according to several studies. Patients with symptoms that indicate DVT should be questioned whether they inject recreational drugs, and if they do, they should be assessed as high-risk patients [14]. Cooke et al. discovered that using Wells' criteria to classify patients in the emergency room into high, moderate, and low-risk categories (prevalence of DVT 58.3%, 8.9%, and 1.5%, respectively). The SimpliRED D-dimer assay has a sensitivity of 63.4% and a specificity of 74.8%, with a likelihood ratio of 2.52 for a positive test and 0.49 for a negative test. Clinical risk stratification separated patients into three risk groups, high, moderate, and low, but with less discriminating power than Wells anticipated. When used regularly in a crowded emergency room, the SimpliRED D-dimer assay's poor sensitivity throws significant doubt on its ability to rule out DVT, especially in low-risk patients [103].

While screening for DVT in an intravenous drug user setting has not been thoroughly investigated, the possibilities for screening for DVT in other frequent patient groups suffering from DVT have been explored with varying degrees of success. For example, Michetti et al. discovered that trauma patients released to an inpatient acute rehabilitation center (ARC) had a low incidence of occult DVT (4.8%) on routine screening duplex done on admission in one study. In addition, ICU length of stay, older age, fewer days on chemoprophylaxis, and delayed chemoprophylaxis initiation have all been identified as risk factors for DVT, and while this study does not support broad screening of trauma patients upon admission to ARC, selective screening based on defined risk factors could be considered [104].

While Duplex ultrasonography has high sensitivity and specificity for identifying symptomatic proximal DVT, it has poor sensitivity and specificity for detecting asymptomatic DVT in high-risk patients and individuals with isolated calf vein thrombosis, according to research published in 2001. Despite these limitations in particular groups, the use of the vascular laboratory to screen for acute DVT in all patients is gradually rising, even as reimbursement and the number of technicians available to conduct these exams are declining. To ensure optimum vascular laboratory use, national standards in clinical pathways based on literature evidence must be established and implemented [105].

In 2007, Furlan et al. predicted that weekly DVT screening during the first 13 weeks following a spinal cord injury (SCI) might identify most asymptomatic DVT episodes when they were still in the acute stage. As a result, this approach may have a long-term effect on the morbidity and mortality associated with VTE after severe SCI. In addition, D-dimer, ultrasonography, and magnetic resonance venography were also shown to be viable DVT screening tests [106].

All patients suspected of having DVT who were IVDU tested positive for DVT on Doppler ultrasound scans, according to research findings, including 109 patients who had DVT examinations. Intravenous drug abusers were also more likely to be admitted to hospitals as a group for investigation and treatment, and the kind and length of therapy received differed [14].

Since this issue has not been well researched, we would want to conclude that further research is needed to examine the relative benefits of screening for DVT in IVDU. Sonography, however, is the recommended technique for evaluating suspected lower extremity DVT. Although it is quite accurate in symptomatic patients for the deep thigh veins, it is less reliable in the calves and pelvis. Combined CT venography and pulmonary angiography enables a more thorough evaluation of PE and DVT in patients with suspected PE, and it offers many benefits than CT pulmonary angiography (CTPA) alone. MRI may be utilized to solve problems in specific situations. The newest radiopharmaceutical, Tc99m apcitide, may make it easier to distinguish between acute and chronic CT, which is challenging to do with other modalities. Traditional venography is of historical significance as a diagnostic technique. However, it is still utilized for anatomical delineation before inferior vena cava (IVC) filter installation as a road-mapping method. Venography of the lower and upper limbs is often used with DVT therapy options, including thrombolysis, percutaneous thrombectomy, angioplasty, and stent insertion [107].

Treatment

For patients who tested positive for DVT, most physicians would select between low-molecular-weight heparin (LMWH) and oral anticoagulation. Because there is not much research on the optimal therapy for an IVDU with DVT, current recommendations are based on local standards. Low-molecular-weight heparin is a safe and straightforward anticoagulant to use. Patients using LMWHs also need less regular blood monitoring than patients taking warfarin; in Sheffield, the local strategy has been to check a complete blood count once a week to screen for LMWH problems. However, the length of therapy may affect a clinician's prescription, and it is in this case, a balance between risk and benefit must be maintained. To minimize the risk of problems such as bleeding, an IVDU therapy may be shorter than that of a non-IVDU; nevertheless, shorter treatment regimens increase recurrence and morbidity [14].

The use of low-molecular-weight heparin for IVDU with DVT was investigated in research published in 2004 by Russell et al. LMWHs are a safe and effective therapy for DVT, according to the researchers. In addition, the medication is a recognized alternative to warfarin in some patient populations, such as pregnant women. With warfarin, doctors can maintain track of the anticoagulant's efficacy by monitoring the international normalized ratio (INR) in the clinic. However, there is no way to tell whether a patient is taking the medication with LMWHs, which is a worry, given that IVDU has a reputation for being a volatile patient group. In addition, no randomized controlled trials (RCTs) are comparing LMWHs and coumarins in the treatment of intravenous drug users. Consequently, there are currently very little data on the optimal methods for managing this patient population [108].

Low-molecular-weight heparin is an excellent antithrombotic drug that does not need to be monitored when used correctly. However, for various reasons, the planned treatment period may not be met, and the risk of bleeding in this group is considerable. As a result, the patient should be told that the LMWH therapy should be maintained for another 12 weeks. In ambulatory settings, accurate assessment of compliance with LMWH indications is complex, and one research showed that treatment lasted between 2 and 12 weeks (on average, 6.5 weeks) [43].

Outpatient therapy for confirmed DVT is currently offered in the majority of UK emergency rooms. According to research performed in Sheffield in 2004, admitting or managing IVDU should be decided entirely based on the clinical situation, not the drug user [14]. Intravenous drug abusers are a patient population prone to prejudice and treatment bias, and doctors must prevent such discrimination and offer the best treatment possible. According to nursing studies, views about IVDU differ by clinical grade, with senior professionals having a more positive attitude toward IVDU. The kind of therapy given to our IVDU group at follow-up visits was similarly inconsistent. According to Lawson et al., getting the right length of treatment after a hospital stay may be difficult. A 16-day course of treatment was the norm in one institution. The authors recommend six weeks of monitored LMWH usage in IVDU as a sensible approach to antithrombotic therapy [108].

Other problems include a lack of therapeutic effectiveness indicated by an unsatisfactory INR value and poor adherence to the reasons for warfarin therapy. In a small sample of individuals, just 20% finished a six-month warfarin treatment cycle. Patients who were given LMWH had a higher level of compliance [109]. It is essential to consider placing a filter into the inferior vena cava if there is an increased risk of serious hemorrhagic complications during antithrombotic therapy [43]. Compression treatment, including graded compression stockings, should be given the same way for the general population [34]. When prescribing therapy to this population of individuals, it seems that a balance of risks and benefits must be evaluated. Shortening the overall length of treatment in IVDU may be helpful, but only if the acute phase is followed strictly and the higher risk of complications and disease recurrence is understood. Addiction treatment clinics that provide supervised thrombosis therapy with LMWH in addition to methadone/buprenorphine administration should be recommended to patients [110-111]. There is presently no information on the usage of new oral antithrombotic medicines in IVDU with DVT. DVT therapy is now regulated by general population guidelines, which means that when antithrombotic medication is given to IVDU, all of the risks and restrictions described above must be taken into account [112]. In addition, local DVT treatment guidelines must be established for these individuals [15].

Prevention

Patients with a habit of injection drug use must be educated about venous problems, and access to proper medical treatment must be made easier [45,110]. Active IVDU, for example, may get training at supervised injection facilities or supervised substitution therapy clinics.

Every physician taking care of IVDU should advise them on reducing health risks, overcoming addiction, preventing intravenous drug administration, or using safe injection techniques if the latter is not feasible [45].

The scope of harm reduction knowledge includes information on medical complications associated with intravenous narcotic use, indications for safe and hygienic injection methods, proper drug filtering, the use of single-use equipment, hand washing, alcohol skin sterilization, and vein puncture techniques. The development of harm reduction services and the distribution of single-use equipment, cleaning and sterilizing agents, and filters are critical [45,113-115].

Complications

Multiple complications can develop after DVT in IVDU such as septic DVT (SDVT), post-thrombotic syndrome (PTS), venous leg ulcer (VLU), and chronic thromboembolic pulmonary hypertension (CTEPH) [115,116].

Septic DVT

SDVT is one of the life-threatening complications affecting large proximal veins commonly secondary to *Staphylococcus aureus* infection. It is more prevalent in frequent long-term IVDU, which can be confirmed by helical CT or color-coded Doppler ultrasound [115]. Manifestations include fever, chills, rigors, and draining sinus with further life-threatening complications such as septic PE and right-sided infective endocarditis [35]. The mainstay of treatment is intravenous b-lactamase-resistant penicillin [115]. Other treatments aimed at preventing further embolization and removing thrombus include catheter-directed thrombolysis, mechanical thrombectomy, surgical thrombectomy, and phlebectomy [35].

Post-thrombotic Syndrome

PTS, formerly known as postphlebitic syndrome, can occur in 20% to 50% of DVT patients despite anticoagulant treatment manifesting as ipsilateral venous insufficiency with pain, heaviness, swelling, skin pigmentation, and venous ulceration within three to six months up to two years or longer after DVT mainly due to venous hypertension, inflammation, and genetic predisposition to vein wall remodeling [36,115,117-121]. The most common risk factors include obesity, older age, proximal DVT, and a history of previous ipsilateral DVT and a supervised exercise program will improve symptoms [120-122]. PTS causes loss of mobility and significantly reduces the patient’s quality of life [115,117]. Clinical diagnosis is used to establish PTS as there is a lack of a gold standard test [123]. The severity of PTS in adults can be measured using the Villalta score [124].

Venous Leg Ulcer

VLU, also known as stasis ulcer, may be associated with severe PTS and is seen in at least 6% of DVT patients even after compression bandaging treatment [115,117,125]. In general, the prevalence of VLU in western countries ranges between 1% and 5% [126-127]. VLU is secondary to inflammation, which involves intracellular edema, endothelial damage, leukocyte activation, and platelet aggregation. DVT is the most common risk factor for VLU. Other risk factors may include advanced age, obesity, and a history of leg trauma [128]. The major clinical manifestation of VLU is an irregular and shallow ulcer on bony prominences with subsequent risk of complications such as cellulitis, osteomyelitis, and malignant transformation. There are various treatment options including leg elevation (minimizes edema), compression therapy (improves blood return), wound dressing (aids healing), pentoxifylline (anti-inflammatory and vaso-dilator), aspirin (anti-inflammatory and anti-platelet aggregation), and surgery in the case of large, prolonged, or refractory VLU [128].

Chronic Thromboembolic Pulmonary Hypertension

CTEPH is a group four pulmonary hypertension (PH) secondary to pulmonary artery obstruction from unresolved thromboembolic disease manifesting as a late complication after PE and DVT [129]. A large population-based cohort study in England on CTEPH revealed an incidence rate of 3.5 per 1000 person-years. The cumulative incidence of CTEPH at 2 and 10 years were 1.3% and 3.3% following PE and 0.3% and 1.3% following DVT, respectively [130]. Risk factors for CTEPH include age >70 years, female gender, PE at first VTE, subsequent PE, chronic obstructive pulmonary disease (COPD), cardiac failure, and arrhythmia such as atrial fibrillation [130]. The main clinical features include progressive dyspnea and exercise intolerance but occasionally can present with right ventricular dysfunction, such as peripheral edema, exertional chest pain, syncope, or near-syncope [131-132]. The multi-step-diagnostic approach includes a high index of suspicion in those with symptoms of dyspnea and/or exercise intolerance after a history of VTE (PE and DVT), measuring pulmonary pressure, and identifying lung clots which can be achieved using a diagnostic algorithm [133]. Some CTEPH diagnostic modalities include echocardiography, pulmonary function testing, ventilation/perfusion (V/Q) scanning, CT pulmonary angiography (CTPA), right heart catheterization (RHC), and contrast pulmonary angiography [134-135]. Pulmonary thromboendarterectomy (PTE) sometimes referred to as pulmonary endarterectomy (PEA) is currently effective and curative treatment for CTEPH [136]. The prognosis of patients with CTEPH is poor without intervention [137-138].

Outcome measures

Intravenous drug abusers with DVT have poor outcomes with high morbidity, mortality, and healthcare costs as compared to the general population. Various studies found that they have slower recovery, recurrent VTE, longer hospital stay, high readmission rate, and low survival thus, negatively impacting productivity and the overall economy.

Recovery

Normally, it takes weeks to months for complete recovery from DVT or PE [139]. However, IVDU-DVT patients have slow recovery and longer hospital stays once admitted for inpatient investigation and treatment [14]. Their recovery is limited by the poor physical and psychological health status on top of the chaotic drug-use lifestyle [6,14].

Recurrence

Studies show that 2%-5% of people experience DVT in their lifetime with increased risk as age advances and there is a high incidence of DVT recurrence after the first attack [140]. Recurrent venous thromboembolism (VTE), PE or DVT, are well-documented complications of DVT [116]. After initial DVT, there is an 8% chance of developing further DVT or PE over one year [141-142]. A retrospective cohort study by Farzamnia et al. identified IV drug abuse as one of the predictive factors for recurrent DVT [140].

Readmission Rate

A retrospective analysis of US databases by Spyropoulos and Lin showed the hospital readmission rates within 90 days were 50.7% for DVT and 58.6% for PE. The same study revealed the readmission rate within one year for the combined diagnoses (DVT or PE) was 5.3% for primary and 14.3% for secondary diagnoses [143].

Survival

Heit et al.'s study on predictors of survival after VTE demonstrated a dramatic decline in survival from day one to over the period of eight years: 97.0% vs. 65.2 % for DVT and 63.6 % vs. 34.5 % for PE [144]. The presence of other comorbidities in IVDU predictively lowers both short and long-term survival.

Mortality

There is an increased risk of dying from VTE in the long term. Mortality of DVT and PE within one month is approximately 6% and 12%, respectively [145]. In another study, a 6% mortality rate has been reported in the first six months after DVT onset [146]. The eight-year mortality risk of VTE (DVT and PE) was reported as 12% by a recent study [147]. A population-based cohort study on VTE adjusted mortality rate ratio (AMRR) within 1 to 10 years and 11 to 30 years of follow-up found a 3-5x increase after DVT and a 6-10x increase after PE [148].

## Conclusions

Intravenous drug use is an epidemic contributing to a wide array of the poor physical, psychological, social, and economic well-being of millions throughout the world of which DVT is one of them. Intravenous drug use is a well-documented risk factor for DVT, which is also increasing at an alarming rate. Indeed, scientific communities are aware of the prevalence, sociodemographic factors, and complications of DVT in IVDU. However, the similarities in presentation between DVT and a variety of other conditions may contribute to it being overlooked and undetected in IVDU by physicians at large. While there are no well-established standard guidelines for the screening and management of DVT in IVDU, the authors recommend six weeks of monitored low-molecular-weight heparin (LMWH), such as enoxaparin at 1 mg/kg SC q12hr along with initiating warfarin therapy within 72 hours of starting LMWH. Continue to administer enoxaparin for five to seven days and until a therapeutic oral anticoagulant effect has been achieved (INR 2.0-3.0). Additionally, clinicians who encounter IVDU patients should counsel them on ways to minimize health risks, overcome addiction, avoid intravenous drug administration, and use safe injection techniques to avoid DVT. Further research is needed to gain a better understanding of the problem, assess the relative benefits of DVT screening in IVDU, and establish preventable measures. More attention by medical associations and governmental bodies should be directed toward developing a set of guidelines or protocols that address screening and management of DVT in IVDU.
